# Michigan State Board of Health

**Published:** 1879-11

**Authors:** 


### ARTICLE XII.

### MICHIGAN STATE BOARD OF HEALTH.

The regular quarterly meeting of this board was held at its office in Lansing, on Tuesday, October 14, 1879, a full board being present. The board is as follows: Prof. R. C. Kedzie, president; H. O. Hitchcock, M.D.; Hon. Leroy Parker; D. C. Jacokes, D.D.; Henry F. Lyster, M.D.; John H. Kellogg, M.D., and Henry B. Baker, secretary.

Dr. Kedzie read a paper on the

### "WASTE OF HUMAN LIFE,"

based principally on the recent Adrian and Jackson horrors. The paper stated in strong terms the necessity for some more efficient preventive measures, and by his request the subject was referred to the committee on legislation with instructions to report in regard to existing laws, the necessity for additional legislation, and, if necessary, incorporate a bill with its report.

### LOCATION OF CEMETERIES.

Dr. Hitchcock presented the subject of the proposed location of a cemetery within the incorporated village of Richland. No formal action was taken, but the members seemed agreed that cemeteries should not be located in villages.

Dr. Baker presented the request of the recorder of Caro, Tuscola county, that he visit and examine into the sanitary condition of the place and the cause of the spread of diphtheria. The board ordered the secretary to comply with the request, if the village authorities pay the expense of the trip, as the small appropriation made by the legislature to the board is exhausted.

The secretary reported several circulars relative to the duties of health officers, local boards of health, and others, which have been prepared, but not issued on account of lack of funds. He was authorized to publish them in the annual report for 1879, and, if possible, to have a large number of reprints printed for distribution to health officers and members of local boards of health.

### ACUTE GLANDERS.

The secretary read a report of a fatal case of acute glanders, reported by S. P. Duffield, M.D. A young man residing in Wayne county purchased a horse in Detroit, afflicted with glanders. The young man took the disease from the animal and died a horrible death. The secretary read a report of a similar case, occurring in Troy, Oakland county, reported by Dr. J. A. Post. He also read an abstract of a paper on the subject of glanders, mentioning other cases which have been reported to the board. This paper embodied a description of the disease, the method by which it is communicated, suggestions for its prevention by means of a prompt report of cases, the isolation of animals or persons afflicted, the destruction of animals, disinfection of surroundings, the existing laws in this State, the laws in other States, and a proposed series of regulations to be adopted by local boards of health in Michigan.

A communication from Dr. O. Marshall, of Lansing, was read relative to diphtheria in the township of DeWitt, Clinton county. A map accompanied this paper, showing the number of cases and the deaths at each house, the order of the occurrence of cases, and the paper showed the methods by which the disease was communicated from one person to another. He was able to trace such communication in nearly every case.

ANNUAL REPORT OF SECRETARY.

In accordance with the by-laws of the board, the secretary presented his annual report of the property of the board, which included the additions to the library (now numbering 1,769 entries, instruments purchased, etc).

The amount of expenditures of the State board of health for the fiscal year ending Sept. 30, 1879, was reported as follows:

| | | |
|---|---|---|
| Chemical analyses | | $ 10.00 |
| Engraving, drawing, etc | | 180.00 |
| Expenses of members | attending meetings | 150.98 |
| | other official | 189.93 |
| Instruments and books | | 173.62 |
| Paper, stationery, etc | | 243.47 |
| Postage | office | 652.39 |
| | members | 15.28 |
| Printing and binding | | 659.60 |
| Secretary | | 2,000.00 |
| Special investigations | | 41.40 |
| Miscellaneous | | 93.39 |
| Total | | $4,410.06 |

NATIONAL HEALTH SERVICE.

A communication was received from the national board of health, asking the advice of the board as to the best plan for the organization of a national health service, and a plan prepared by Dr. Hitchcock by request of the board, was adopted, and the secretary directed to transmit it to the national board of health.

QUARTERLY REPORT.

The secretary read a report of the work of the office since the last meeting. During this time the correspondence with local boards of health, observers of diseases, and meteorological observers has been kept up, and nine new meteorological stations have been established. Over 500 pages in the letter-book have been used in copying correspondence, and a large number of communications have been received and passed over to appropriate committees. Fourteen hundred copies of the sixth annual report have been wrapped, directed and distributed to health officers and others. Correspondence relative to sanitary appliances has been had, and four articles have been added to the exhibit in the office of the board, namely, an Ely sewer stench-trap for use on street corners, manufactured by A. Ely, 110 West ave., Rochester, N. Y.; a Parmenter's air-moistener, and a furnace air-moistener, manufactured by I. W. Parmenter, 15 Murray St., N. Y. City; and a sample of a new disinfectant called Little's soluble phenyle, for sale by T. W. Lawford, 10 S. Holliday St., Baltimore, Md.

### STEAMBOAT INSPECTION.

The secretary presented the subject of the necessity for steamboat inspection on the inland lakes of the State, and read correspondence from James A. Dumont, U. S. supervising inspector-general of steamboats. Mr. Dumont says that "any State having navigable waters is guilty of a crime against its citizens every day that it fails to enact laws for their protection when on steamers navigating such waters." This subject was referred to the Hon. Leroy Parker, committee on legislation, who was asked to consider the question of making the U. S. regulations for steamboats applicable to the inland waters of Michigan.

A report was made of the correspondence with the Grand Rapids board of health, relative to the office and salary of health officer in that city. Mr. Parker had prepared, at considerable labor, an opinion on the subject, which has been submitted to, and approved by, the attorney-general.

### SANITARY PROTECTION.

Dr. J. H. Kellogg read a report on "sanitary protection associations in cities and villages." He presented strong arguments for a sanitary association in every city and village in the State, which shall coöperate with the local boards of health, wherever possible, and secure action when they are inefficient. He gave a detailed plan for the organization of such associations.

### SANITARY CONVENTIONS.

Drs. Hitchcock and Lyster, of the special committee, reported details of plan for a sanitary convention on January 7, 8, next, at Detroit, and one at Grand Rapids during the month of February, 1880.

THE CONVENTION AT DETROIT

Will be held in St. Andrew's Hall, on the dates named above. There will be two sessions each day from 9 a. m. till 12 m., and from 2 p. m. till 5:30 p. m. The officers of this convention are as follows: President, Ex. Gov. Hon. H. P. BALDWIN; Vice-Presidents, Hon. JAMES BIRNEY, U. S. Minister to the Hague; WM. BRODIE, M.D., Hon. WM. L. WEBBER, Mrs. JOHN J. BAGLEY, and Mrs. MORSE STEWART; Local Secretary, C. HENRI LEONARD, M.D., 50 LaFayette Ave., W. Detroit, Mich.

SUBJECTS TO BE PRESENTED.

1. Abattoirs for cities.
2. School hygiene.
3. Ventilation of living and sleeping rooms.
4. Cooking schools.
5. Plumbing for dwellings.
6. Prevention and limitation of contagious diseases.
7. Inspection of food.
8. Water supply for the family.

CONVENTION AT GRAND RAPIDS.

The minor details of this convention are not perfected, but these and the date will be announced hereafter. The president of the convention will be Rt. Rev. Geo. D. Gillespie, and the local secretary will be Arthur Hazlewood, M.D.

SUBJECTS TO BE PRESENTED.

1. Public interest in, and importance of, general sanitation.
2. School architecture in respect to its hygienic as pectsand importance.
3. Sewerage—its importance, its benefits, and its dangers.—E. H. Van Deusen, M.D.
4. Sanitation of the sick-room.
5. Infection. The every-day dangers of it, and how to prevent it.
6. Habits in their relation to health and disease.

There will be an evening session from 7 till 9:30 o'clock at the opening of the convention ; on the following morning a session from 10 a. m. till 12 m ; a session from 2 p. m. till 5 p. m., and an evening session from 7 till 9:30.

### EXHIBITION OF SANITARY APPARATUS.

Manufacturers of all kinds of sanitary apparatus or appliances are invited to send specimens of their manufactures for exhibition at these conventions in accordance with the following regulations:

(*a.*) The Board of Health reserves the right to decline any article not deemed suitable.

(*b.*) A full description of each article proposed to be exhibited must be forwarded to the Secretary of the convention with the application for space.

(*c.*) There will be no charge to exhibitors for entrance fee or for wall or floor space.

(*d.*) Exhibitors will pay all expenses of transportation, storage, placing and removal of goods, and must themselves be responsible for any breakage or damage to their articles.

(*e.*) Every article exhibited, and every model, drawing or photograph must bear a descriptive label giving a detailed statement respecting its construction, use and the price at which it can be furnished, and the name and address of the agent and place of sale.

(*f.*) Exhibitors may employ persons to explain their exhibits, and properly to solicit orders.

(*g.*) The position of articles in the hall will be determined by the Secretary of the convention.

(*h.*) Articles for exhibit will be received by the Secretary of the convention at Detroit, Dr. C. H. Leonard, 50 La Fayette avenue, west, from December 15, 1879, to Jan. 6, 1880. The time of entering articles at Grand Rapids has not been determined.

### JUDGES, AWARDS, ETC.

Competent judges will be invited to thoroughly examine the articles exhibited and certificates of merit will be awarded. The records of the proceedings, papers and addresses, and a catalogue of articles exhibited with award of the judges will probably be published in the annual report of the Secretary of the State Board of Health. Reprints from that of the proceedings may contain several pages of advertisements for which there shall be charged the following rates, $10 per page, or $6 per half page, the payment of which secures the advertiser ten copies of the reprint, and additional copies at cost.

The admission to the exhibit and to all sessions of these conventions shall be free.

### A PLAN FOR EXAMINATIONS.

Dr. Lyster, special committee on proposed examinations by this board in sanitary science, reported a plan. Dr. Baker proposed some slight modifications, and the plan was adopted. It contemplates the examination and granting of certificates to such persons as sustain an examination showing them qualified to act as health officers. The first examination will be held in July next.

### DISEASES AND SLAUGHTER-HOUSES.

Dr. Hitchcock presented a voluminous report on epidemic, endemic and contagious diseases, mainly relating to the subject of diphtheria; also, a report on slaughter-houses, rendering establishments, etc. In this paper he recommended and proved the economy of confining this business to one establishment in each city and village.

### "DROWNED" LANDS.

Dr. Lyster presented an exhaustive paper on the reclamation of "drowned" lands, showing their great influence on public health. In this paper he includes a translation of a paper relating to the influence on health of "drowned" lands and their reclamation in a district in France, the facts covering a period of 20 years.

### VENTILATION.

Dr. Jacokes presented a paper on "warming and ventilating private dwellings and public buildings already constructed."

His paper contains diagrams illustrating the methods recommended.

After the transaction of a large amount of business, the board adjourned. The next regular meeting of the board will be on Tuesday, January 13, 1880.

---

GRATIFYING PROGRESS.—The first medical school adopting the full graded system of medical instruction in this country was the Chicago Medical College, organized in this city in 1859. For twelve years it stood alone. In 1871, the medical school of Harvard adopted the same system. The medical department of the new Syracuse University, in Syracuse, N. Y., followed about the same time. Within the last two years the University of Pennsylvania, the medical department of the University of Michigan, the medical department of the Iowa University, and the medical department of Yale have entered upon the same course. And now we have an official announcement that after the close of the present college term the Bellevue Hospital Medical College, in New York, and the Detroit Medical College, Mich., will adopt the full three years, graded system ; and the authority of the St. Louis *Clinical Record*, that the St. Louis Medical College has also adopted the same course. Truly the cause of progress is onward, and with such increasing force as to make the long-desired revolution in medical teaching complete in a very brief period of time. D.

TRI-STATE MEDICAL SOCIETY, OF INDIANA, ILLINOIS AND KENTUCKY.—The fifth annual meeting of this society will commence on the morning of Tuesday, November 4th, in Evans Hall, Evansville, Indiana, and is expected to continue four days. The programme contains many interesting topics, and the president, Dr. J. A. Ireland, of Kentucky, is to deliver a public address on the evening of the first day of the meeting. Many of the railroads connecting with Evansville, will carry members of the society at reduced rates.

